# The Regnase pathway: a core axis in immune regulation and inflammatory disease

**DOI:** 10.3389/fimmu.2026.1756491

**Published:** 2026-02-27

**Authors:** Luca Muzio, Davide Ferrati, Eleonora Colombo, Claudia Molinaro

**Affiliations:** Neuroimmunology Unit, Department of Neuroscience, Istituto di Ricovero e Cura a Carattere Scientifico (IRCCS) Ospedale San Raffaele, Milan, Italy

**Keywords:** cytokine homeostasis, immune tolerance and inflammation, post-transcriptional regulation, Regnase-1, RNA-binding proteins, ZC3H12A/MCPIP1

## Abstract

The Regnase/MCPIP ribonucleases are involved in the regulation of immune homeostasis, by degrading RNA transcripts that encode inflammatory and regulatory proteins. This review highlights their molecular architecture, catalytic mechanisms, and intricate regulatory networks that orchestrate innate and adaptive immunity. This article presents a narrative review of the literature on distinct physiological roles of individual family members and how their dysfunction drives inflammatory, autoimmune, fibrotic, metabolic, and neoplastic disorders across multiple tissues. Although Regnase family members exhibit some functional redundancy, each also possesses distinct, non-overlapping roles. Regnase-1 restrains cytokine production and along with Regnase-2 modulates neuroinflammation. Both Regnase-3 and Regnase-4 possess homeostatic functions although they are also involved in orchestrating interferon and myeloid signaling and contribute to immune regulation and tumor suppression. We also examine emerging therapeutic strategies targeting Regnase activity, including antisense oligonucleotides to enhance Regnase-1 expression, gene- and RNA-based delivery approaches, and selective inhibition of Regnase-1 in T cells to boost cancer immunotherapy. Together, these findings underscore Regnase proteins as central post-transcriptional checkpoints in immunity and highlight their potential as targets for treating autoimmune disease, chronic inflammation, fibrosis, and cancer.

## 1 Introduction

For many years, the complexity of immune regulation was primarily attributed to transcriptional networks controlling the expression of cytokines (e.g. Interleukin-6, IL-6), chemokines and transcription factors (TFs) such as Nuclear Factor kappa-light-chain-enhancer of activated B cells (NF-κB) (1, 2). However, growing evidence demonstrates that post-transcriptional regulation of messenger RNAs (mRNAs) plays an equally crucial role in shaping immune responses (3).

Translational control modulates protein synthesis and therefore the plasticity of the immune system (4). In addition, some factors (e.g., AU-rich element-ARE–binding proteins and miRNAs) can destabilize mRNAs, thereby reducing the amount of transcript available for translation. The mRNA degradation provides immune cells with a rapid mechanism to adapt to environmental cues by dynamically regulating transcript abundance.

Ribonucleases (RNases) mediate the processing, decay, and quality control of mRNAs (5), while cis-acting sequence elements—mainly located within 3′ untranslated regions (3′ UTRs)—and their interaction with RNA-binding proteins (RBPs) add a further layer of specificity to RNA fate determination (4). Beyond AREs, mRNAs can harbor stem–loop structures that act as anchoring elements, facilitating the interaction with RBPs (6). They are three-dimensional structures that allow the interaction of target mRNAs with RBPs. Stem-loop structures comprise pyrimidine-purine-pyrimidine tri-loop sequences often located within the 3′ UTR of inflammatory mRNA that are recognized and bound by RBPs, such as Regnase-1 (7).

However, RBPs functions go beyond the simple RNA degradation as they serve as a central hub of post-transcriptional control, coordinating RNA splicing, transport, localization, stability, and mRNA translation (8). In immune cells, they play indispensable roles in balancing activation and tolerance by ensuring transient expressions of cytokines, chemokines, and TFs that orchestrate both pro- and anti-inflammatory signaling (9). By recognizing these sequences and structural elements within 3′ UTRs of target mRNAs, RBPs can either stabilize or promote the degradation of transcripts. Disruption of this finely tuned balance usually leads to cytokine overproduction and immune dysregulation, driving chronic inflammation, autoimmunity, as well as a general loss of immune tolerance (10, 11).

Historically, researchers focused on non-catalytic RBPs such as Tristetraprolin (TTP, encoded by the *Zfp36* gene (12),), HuR [encoded by *Elavl1* (13)], and Roquin [encoded by *Rc3h1* (14)], which regulate mRNA decay indirectly by recruiting enzymatic complexes that remove the mRNA’s poly(A) tail (deadenylation) or the 5’ cap (decapping) (15). A landmark study in 1998, by Carballo and colleagues, demonstrated that TTP binds ARE sequences located within the 3′ UTR of Tumor Necrosis Factor (TNF)-α mRNA, and promotes its degradation in macrophages—thus establishing the concept of ARE-mediated decay as a key immunoregulatory mechanism (16, 17).

Following these pioneer findings, the field expanded rapidly, and additional RBPs were subsequently identified. Indeed, in 2006, the discovery of Regnase-1 (encoded by *Zc3h12a* gene and also known as Monocyte Chemoattractant Protein-1–Induced Protein 1, MCPIP-1) (18) introduced the concept of enzymatic RBPs, which are proteins that both recognize RNA and catalyze its degradation through intrinsic RNase activity (19, 20). Two years later, the same research group identified three similar paralogs, establishing a new gene family with conserved catalytic and regulatory features. The initial name MCPIP-1, assigned to the first protein of the family, derives from the observation that its activation involves the monocyte chemoattractant protein-1 (MCP-1), which is known chemoattractant responsible for the migration of monocytes/macrophages and involved in inflammatory processes (18).

**Figure 1:**
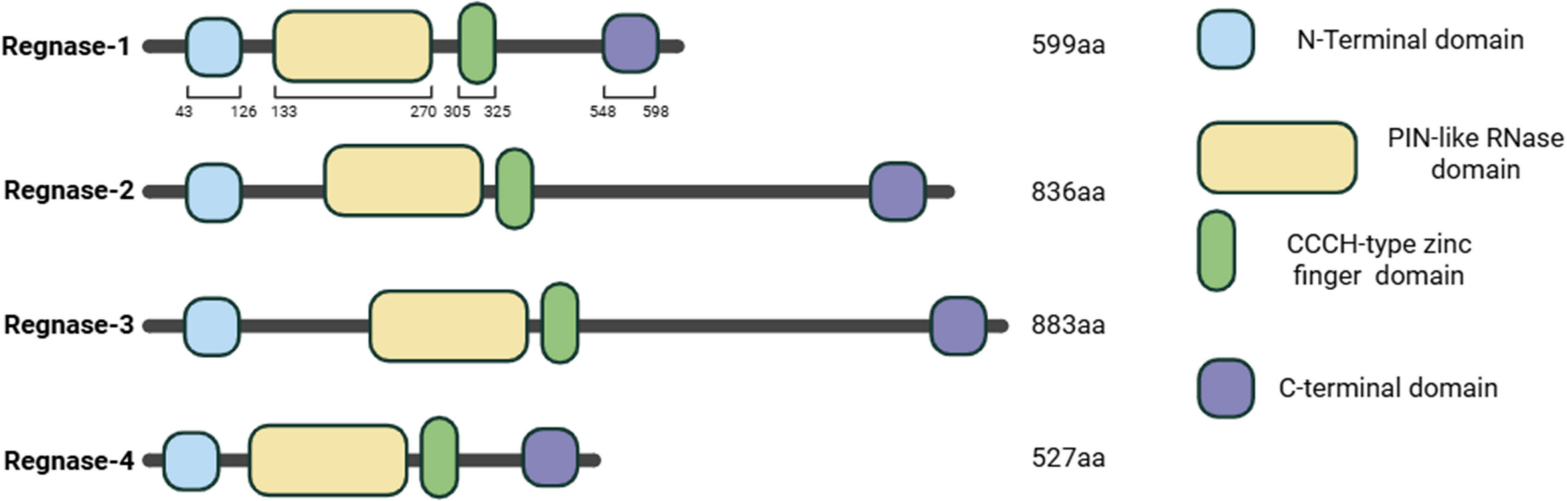
Conserved domain organization of the Regnase protein family. Schematic representation of the structural architecture of the four mammalian Regnase paralogs: Regnase-1 (*ZC3H12A*, 599 aa), Regnase-2 (*ZC3H12B*, 836 aa), Regnase-3 (*ZC3H12C*, 883 aa), and Regnase-4 (*ZC3H12D*, 527 aa). Despite differences in total protein length, all family members share a conserved core domain organization composed of: (i) an N-terminal domain (NTD), (ii) a centrally located PIN-like RNase (NYN) catalytic domain responsible for endonucleolytic cleavage, (iii) a CCCH-type zinc-finger domain that mediates RNA substrate recognition, and (iv) a C-terminal domain involved in regulatory and protein–protein interactions.

The *Zc3h12* gene family (human orthologs *ZC3H12A–D*) derives its name from “Zinc finger CCCH-type containing protein 12,” referring to the defining CCCH-type zinc-finger RNA-binding motif common to all members. Although most zinc fingers domains are generally regarded as DNA binding, the CCCH-type subfamily, among others, preferentially binds and modulates RNAs (21). Each paralog (*Zc3h12a–d*) encodes Regnase-1 through Regnase-4, respectively, reflecting their evolutionary relatedness and shared function as RNA-degrading enzymes. Each Regnase protein contains two conserved domains: a CCCH-type zinc-finger that mediates RNA recognition and a N-terminal PilT N–terminus (PIN)-like RNase domain, which catalyzes endonucleolytic cleavage (22, 23), (**Figure 1**). Through this dual-domain architecture, Regnase proteins selectively degrade mRNAs encoding key inflammatory mediators —such as *Il6*, *Il1, Il2*, *Il12b*, and components of the NF-κB pathway— thereby establishing a rapid post-transcriptional feedback loop that constrains immune activation. By coupling RNA recognition with catalytic decay, the Regnase family acts as a central checkpoint safeguarding immune homeostasis. When dysregulated, Regnase activity fails to restrain cytokines production, leading to persistent inflammation, autoimmunity, and tissue fibrosis, as shown in both experimental models and human disease (22, 24).

In summary, Regnase proteins function alongside factors such as Roquin (25), or other ARE–binding proteins, and miRNAs, forming a post-transcriptional regulatory network that precisely shapes cytokine production and immune cell differentiation. In general, their importance lies in their ability to function as molecular “brakes” that balance immune defense and immune restraint, which is essential for long-term immune system homeostasis and health. Objectives of this narrative review are to provide the reader with comprehensive overview about the functioning of Regnase proteins in immune cells, tissues and cancer.

## 2 Structural architecture and catalytic mechanism

The Regnase family is defined by a conserved catalytic architecture that integrates RNA recognition and endonucleolytic cleavage activities within a single polypeptide (23). All four members express an N-terminal CCCH-type ZnF domain, responsible for substrate binding, which is located downstream to a N-terminal PIN-like RNase domain which possesses intrinsic endoribonuclease activity (15, 26, 27). Among members of this family, Regnase-1 remains the most extensively characterized (**Figure 1**).

Regnase-1 is composed of 599 amino acids and encodes a protein with a molecular weight of ~66 KDa (28). Its ZnF domain determines substrate specificity by recognizing stem-loop structures located within the 3′ UTRs of target mRNAs. Early studies proposed that Regnase-1 binds the following ARE motif: “UUAUUUAUU”, but subsequent biochemical and structural analyses revealed that the RNA secondary structure—specifically a short stem followed by a single-stranded loop—is the critical determinant for efficient binding and cleavage (21, 29, 30). This modality of target recognition distinguishes Regnase proteins from other post-transcriptional regulators such as TTP, which binds similar ARE sequences but lack intrinsic catalytic activity and instead recruits external decay protein complexes (31, 32).

The enzymatic activity of Regnase-1 resides within its NYN (Nedd4-BP1, YacP-like nuclease) domain, which adopts a conserved PIN-like protein fold characteristic of metal-dependent endoribonucleases (33). This domain, positioned immediately downstream of the N-terminal domain (NTD) region, harbors the ribonucleolytic catalytic core (20, 34). Within the active site, four conserved aspartate residues—D141, D225, D226, and D244—coordinate a single divalent metal ion (Mg²^+^ or Mn²^+^) which is essential for the catalysis. Among these residues, the D141 has been extensively characterized, and its mutation completely abolishes RNase activity, confirming its indispensable role in coordinating the metal cofactor (20). These aspartate residues form a negatively charged loop that likely contributes to substrate recognition by electrostatically attracting the phosphate backbone of target RNAs. Crystallographic analyses revealed that the NYN/PIN-like domain adopts a canonical α/β fold, precisely orienting these residues for metal chelation and phosphodiester bond cleavage (26, 35). Structurally, Regnase-1 can form homodimers through interactions between its PIN-like domains, an arrangement that enhances substrate processing and mRNA decay efficiency. The R214 residue is critical for this oligomerization. Mutation of R214 in Regnase-1disrupts dimer formation and markedly impairs target mRNA degradation (24). Under physiological conditions, the dimeric form appears predominant, although higher-order tetrameric assemblies have also been reported (29).

Although the NTD of Regnase-1 does not appear to directly contact target mRNAs, it plays a cooperative role in facilitating cleavage together with the PIN-like domain. Experimental evidence indicates that Regnase-1 mutants lacking the NTD fail to produce cleaved mRNA fragments *in vitro*, underscoring the importance of this domain for full catalytic activity of Regnase-1. Nonetheless, the endonucleolytic reaction is executed by the PIN-like domain, as demonstrated by a mutant Regnase-1 variant which contains intact ZnF and NTD domains but a catalytically inactive PIN-like domain (D226N, D244N substitutions) that is unable to degrade target transcripts (24). Sequence homology analyses further reveal that PIN domains are highly conserved among members of the Regnase family (~72% identity), supporting the notion of a shared catalytic mechanism across the family (22, 24, 28). In the ZnF domain members of the Regnase family share percentages of identity are ~74% (36, 37) At the C-terminus, Regnase-1 contains a proline-rich region (PRR) followed by a C-terminal domain (CTD) that confers additional regulatory properties operating beyond the mRNA decay (**Figure 1**). The CTD plays a key role in microRNA (miRNA) recognition and processing, as Regnase-1 can cleave the terminal loops of precursor miRNAs (pre-miRNAs), thereby interfering with miRNA maturation. Notably, this activity is independent of the ZnF domain, since mutation of the CCCH motif (C306R) does not impair pre-miRNA cleavage *in vitro*. Comparative sequence analyses show that the CTD is poorly conserved between invertebrates and vertebrates, suggesting that Regnase-1 acquired anti-miRNA functions later in evolution. Functional studies further support a reciprocal regulatory relationship between Regnase-1 and Dicer in lung cancer cells; indeed, an inverse correlation between their expression levels suggests a delicate balance between productive and abortive RNase activities during miRNA biogenesis (36). Furthermore, Regnase-1-mediated decay of mRNAs often converges with miRNA pathways on the same cytokine transcripts (e.g., *Il6*, *Tnf*). In particular, Regnase-1 directly regulates the miRNA pathway itself by cleaving the terminal loops of pre-miRNAs, thereby suppressing the biogenesis of specific mature miRNAs and establishing a broader layer of post-transcriptional control (36, 38).

Regnase-1 also exerts antiviral activity by targeting viral miRNAs. For example, Kaposi’s sarcoma–associated herpesvirus (KSHV) encodes several miRNAs that are directly cleaved by Regnase-1, reducing their mature levels. However, during latent KSHV infection, Regnase-1 itself becomes downregulated, likely through the action of three specific viral miRNAs that destabilize its mRNA; thus illustrating a viral countermeasure against Regnase-1–mediated RNA surveillance (39).

A defining feature of Regnase proteins is their tightly regulated subcellular localization, which spatially organizes their mRNA decay activity. Members of the Regnase family are predominantly localized within cytoplasm and dynamically partition between processing bodies (P-bodies) and stress granules, two major hubs for mRNA decay, storage, and translational repression (40, 41). This compartmentalization concentrates their enzymatic activity within specialized centers of RNA metabolism, ensuring that degradation occurs in a context-dependent manner.

Regnase proteins also functionally interact with other RNA decay pathways, particularly with Roquin (42), that binds specific cis-elements in target 3’UTR mRNAs, often short stem–loop motifs and then recruits either CCR4–NOT deadenylase complex, promoting poly(A) tail shortening or members of the decapping complex that removes the 5’ CAP of messengers. The cooperation between Regnase-1 and Roquin ensures comprehensive regulation of shared targets: Regnase-1 primarily degrades translating mRNAs, while Roquin targets non-translating mRNAs within P-bodies, providing layered temporal control over the expression of inflammatory mediators (7, 25).

## 3 Functional diversification of Regnase family members

The four Regnase paralogs share a conserved domain architecture that enables targeted mRNA degradation (**Figure 1**). While all members can be recruited to cytoplasmic sites of RNA metabolism such as P-bodies and stress granules, they exhibit distinct expression patterns, regulatory mechanisms, and substrate specificities. This functional diversification allows the family to collectively regulate a broad spectrum of biological processes, from controlling transient inflammatory bursts to maintaining basal tissue homeostasis and cell cycle progression (28). The following sections will delineate the unique and non-redundant roles of each Regnase protein, highlighting how their specialized functions converge to ensure precise post-transcriptional control of immunity and cellular integrity.

### 3.1 Regnase-1 (ZC3H12A/MCPIP1): a regulator of cell activation and the inflammatory cascade

Regnase-1 exerts its primary function as a feedback regulator of inflammatory signaling. It is rapidly induced by the activation of the Toll-like receptor (TLR) downstream cascade, such as IL-1β, and TNF-α, creating an immediate negative feedback loop that degrades key inflammatory mRNAs such as *Il6*, *Il12b*, *Cxcl1*, and the NF-κB subunit *Nfkb1* (20, 43). The activity of Regnase-1 is tightly controlled by post-translational modifications. Upon immune activation, the IκB kinase β (IKKβ) mediates the phosphorylation of Regnase-1 at specific serine residues (S435 and S439 in mice), triggering the recognition of Regnase-1 by the β-transducin repeat-containing protein (βTrCP) and leading to K48-linked polyubiquitination and proteasomal degradation. This cascade results in temporarily relieving Regnase-1-mediated suppressive effects and allows cytokine production (43).

A novel regulatory mechanism involves the Interleukin-1 Receptor-Associated Kinase (IRAK)-1-mediated phosphorylation of Regnase-1 at S494 and S513. The phosphorylation of these residues promotes the binding of Regnase-1 to proteins belonging to the 14-3–3 family. This interaction stabilizes Regnase-1 levels but causes the inactivation of the catalytical domain as well as prevents mRNA binding, thereby contributing to the fine-tuning of the duration of inflammation (43, 44).

Constitutive *Zc3h12a*-deficient mice, which lack Regnase-1, exhibit severe anemia, splenomegaly, lymphadenopathy, and, most strikingly, fatal systemic inflammation by 12 weeks of age, underscoring the physiological importance of Regnase-1 (20, 45). Human genetics studies further confirm the key role of Regnase-1 in immunity; indeed polymorphisms in the *ZC3H12A* gene are linked to autoimmune disease such as ulcerative colitis, where the gain-of-function S438L mutation blocks IKKβ-dependent degradation, leading to excessive protein stabilization and impaired cytokine control (22, 46). Notably, whole-exome sequencing of ulcerative colitis patients has identified recurrent somatic mutations in *ZC3H12* within inflamed intestinal epithelium, further underscoring how dysregulated Regnase-1 activity in epithelial compartments can drive mucosal inflammation and barrier dysfunction (47).

**Figure 2:**
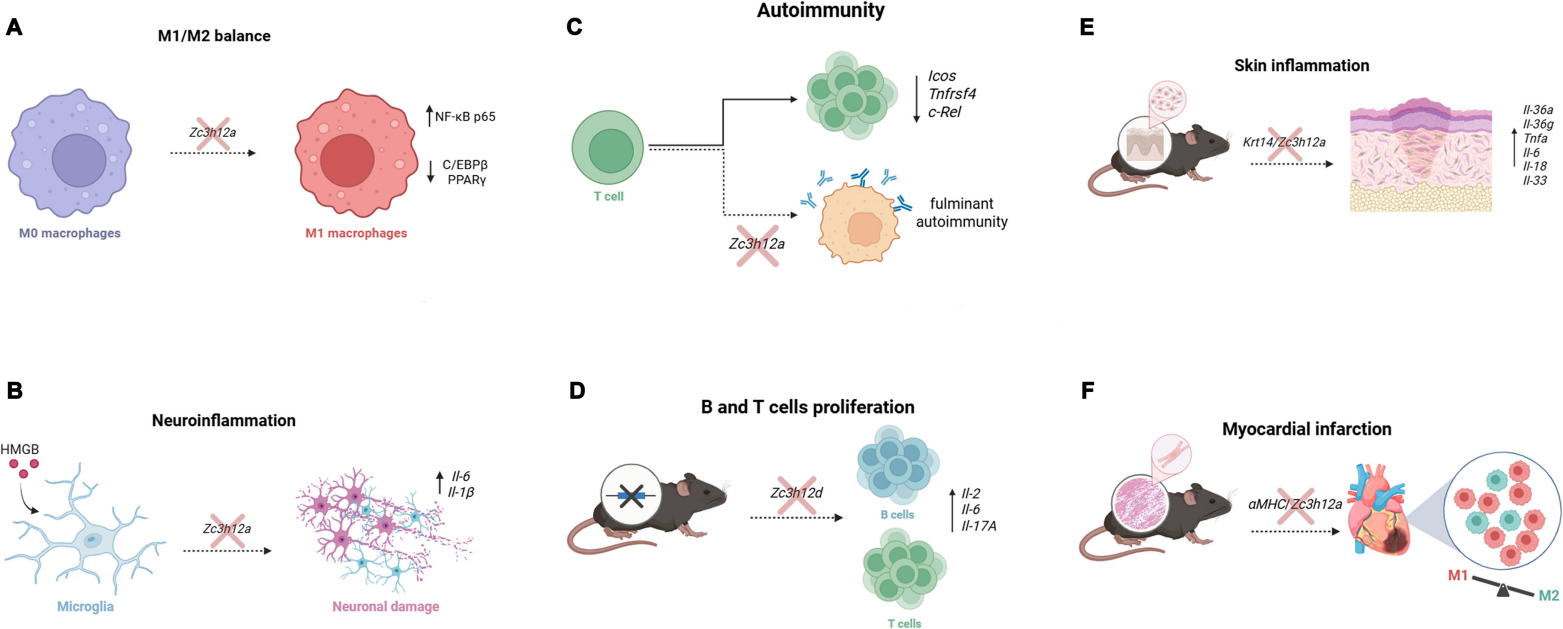
Regnase-family regulation responses across multiple conditions. **(A)** Regnase-1 restrains pro-inflammatory macrophage polarization by degrading target mRNAs and maintaining signaling balance. Loss of Regnase-1 drives macrophages toward an M1 phenotype, enhancing NF-κB p65 activity and reducing C/EBPβ and PPARγ, ultimately amplifying inflammatory responses. **(B)** HMGB1 induces Regnase-1 in microglia, where it limits neurotoxicity by degrading *Il1b* and *Il6* transcripts. Regnase-1 deficiency increases cytokine production and exacerbates neuronal injury. **(C)** In naïve CD4^+^ T cells, Regnase-1 enforces quiescence by constitutively degrading mRNAs encoding key activation factors (*Icos*, *Tnfrsf4*/OX40, *c-Rel*). CD4^+^ T cell–specific deletion of Regnase-1 causes uncontrolled activation, plasma-cell expansion, hypergammaglobulinemia, and fulminant autoimmunity. **(D)** Genetic ablation of Regnase-4 (*Zc3h12d*) leads to hyperproliferation of activated B and T cells and excessive secretion of *Il2*, *Il6*, and *Il-17A*, reflecting failure to degrade inflammatory cytokine mRNAs. **(E)** Keratinocyte-specific deletion of Regnase-1 (*Krt14^Cre^*/*Zc3h12a^flox/flox^*) disrupts cutaneous immune homeostasis. Mutant mice spontaneously develop inflammatory skin lesions accompanied by upregulation of *Il36a*, *Il36g*, *S100a8/9*, *Sprr2* family members, and pro-inflammatory cytokines including *Tnf*, *Il6*, *Il18*, and *Il33*. With age, mice exhibit chronic wounds and localized alopecia, demonstrating the essential role of Regnase-1 in epithelial cytokine control. **(F)** Cardiomyocyte-specific Regnase-1 deletion (*αMHC*^Cre^/*Zc3h12a^flox/flox^*) exacerbates myocardial infarction injury following coronary ligation, resulting in increased inflammation, larger infarcts, reduced survival, and impaired repair. Loss of Regnase-1 skews macrophage polarization toward pro-inflammatory M1 cells—partly via dysregulated p53/ferroptosis signaling—while reducing reparative M2 macrophage responses necessary for cardiac healing.

Conditional knockout models have been employed to elucidate the tissue-specific, non-redundant functions of Regnase-1. Deletion of Regnase-1 in myeloid and granulocyte cells, obtained by a conditional knockout strategy that involves *Zc3h12a*^flox/flox^ mice (48), harboring loxP sites flanking exons 3–5 of the *Zc3h12a* gene, and LysM^Cre^ transgenic mice, which express Cre recombinase under the control of the *Lysozyme 2* (*LysM*) promoter region, resulted in a severe systemic lupus-like autoimmunity, characterized by B-cell expansion, autoantibody production, and inflammatory-mediated damage to the skin, lungs, and kidneys. This occurs because Regnase-1 in myeloid cells acts as a crucial checkpoint of cell activation, and its loss leads to abnormal macrophage activation and the systemic overproduction of inflammatory molecules such as *B-cell Activating Factor* (*Baff*), *Il5*, *Il9* and *Cd40L*, that dysregulate adaptive immunity (**Figure 2A**) (48, 49). Similarly, B-cell–specific deletion of Regnase-1 obtained by crossing *Zc3h12a*^flox/flox^ mice with Mb1^Cre^ (Cd79a) mice leads to severe B-cell hyperactivation, spontaneous germinal center formation, autoantibody production, and systemic autoimmunity, highlighting an indispensable cell-intrinsic role for Regnase-1 in restraining B-cell responses and maintaining tolerance (50).

In the liver, Regnase-1 expression is induced under fibrotic conditions and functions to restrain hepatic stellate cell (HSC) activation. The conditional KO of Regnase-1 obtained by crossing *Zc3h12a*^flox/flox^ mice with *Alb*^Cre^ mice causes the release of connective tissue growth factor, which promotes HSC activation in a paracrine manner, while within HSCs themselves, Regnase-1 inhibits their activation by regulating *Transforming Growth Factor Beta 1* (*Tgfb1*) expression (51).

Conversely, studies involving overexpression mediated by either transgenic or viral approaches reveal that Regnase-1exerts broad, cell-intrinsic anti-inflammatory activities. In macrophages, enforced expression of Regnase-1 negatively regulates c-Jun N-terminal kinase (JNK) and NF-κB signaling by acting as a deubiquitinase for key signaling intermediates, like TNF Receptor-Associated Factor (TRAF)-2, TRAF3, and TRAF6 (52). In adipocytes, retrovirus-mediated overexpression of Regnase-1 shifts the cellular transcriptome and proteome away from a metabolic profile, downregulating factors involved in lipid and carbohydrate metabolism and modulating insulin sensitivity. Similarly, in colorectal cancer cells, restoring Regnase-1 expression by retrovirus inhibits proliferative and migratory capacities by suppressing the NF-κB pathway through negative regulation of K63-linked ubiquitination of TRAF6 (52–54).

Evolutionary analyses highlight the importance of Regnase-1 across species. In zebrafish, the expression of *ZFc3h12a* is tightly regulated during embryogenesis, with transcript levels peaking early in the development and then rapidly decreasing. The perturbation of this expression pattern by microinjecting mRNA encoding for the zebrafish Regnase-1-P2A-mTurquoise protein into the yolks of one-cell stage embryos increases embryonic lethality. However, this phenotype was not observed in controls in which a catalytically inactive Regnase-1 was injected. The overexpression of Regnase-1 causes extensive dysregulation of genes involved in the endoplasmic reticulum stress response, protein folding, and the formation of primary germ layers (55).

In *C. elegans*, the Regnase-1 ortholog (*Rege-1*) modulates senescence-associated lysosomal activity and metabolic pathways, including insulin/IGF signaling (IIS) and the Target of Rapamycin (TOR) kinase signaling, which are also regulated by mammalian Regnase-1 in the context of immunity and metabolism (56). Collectively, evidence from mammalian and invertebrate models establishes Regnase-1 as an indispensable regulator whose function in integrating inflammatory and metabolic signals for immune and cellular homeostasis has been conserved throughout evolution (57).

### 3.2 Regnase-2 (ZC3H12B/MCPIP2): a specialized tissue–expressed RNase

ZC3H12B encodes Regnase-2, an 836-aa protein with an approximate molecular weight of 95 kDa (**Figure 1**) that is the structurally closest paralog to Regnase-1. However Regnase-2 exhibits a more constitutive expression pattern and a greater protein stability, suggesting a primary role in basal RNA turnover rather than in the rapid, inducible regulation of inflammation (58). While it shares the ability to degrade classic inflammatory transcripts, such as *Il6* by interacting with a stem-loop structure in their 3’UTRs, its substrate recognition diverges mechanistically: high-throughput RNA profiling revealed that Regnase-2’s PIN domain, rather than the ZnF domain, mediates RNA binding—an uncommon feature among RNases (59, 60). Regnase-2 is highly expressed in the in the cerebral cortex, hippocampus, and cerebellum, where it functions as a critical homeostatic regulator primarily within glial cells. Indeed, its downregulation in astrocytes leads to increased mRNA levels of pro-inflammatory cytokines such as *Il1b* and *Il6* (61). This role is underscored *in vivo*, where a reduction in Regnase-2 expression during neuroinflammation correlates with elevated cytokine production and enhanced glial activation.

Regnase-2 and Regnase-1 mutually regulate each other by degrading one another’s mRNAs. This balanced opposition is critical for homeostasis, and its breakdown contributes to disease (61, 62). Indeed, Regnase-2 appears to play a role in regulating the turnover of Regnase-1. Inducing the expression of Regnase-2 in glioblastoma cells caused the downregulation of Regnase-1 and such downregulation requires an intact PIN domain, suggesting that Regnase-2 affects the stability of Regnase-1 mRNA (61).

Interestingly, a mutual regulatory loop between Regnase-1 and Regnase1 exists in these cells, as the 3’UTR of Regnase-2 contains sequences that are targeted by Regnase-1. To provide an additional level of regulatory complexity in the fine-tuning of Regnase-2 expression, several ARE sequences that are under the control of TTP have been described in the 3’UTR of Regnase-2 (61).

Regnase-2 restricts not only the repression of a panel of pro-inflammatory signals but operates on cell proliferation and its expression profile in cells is extraordinarily regulated.

### 3.3 Regnase-3 (ZC3H12C/MCPIP3): a specialized modulator of interferon pathways

Regnase-3, encoded by ZC3H12C, is a 883 aa protein with a molecular weight of approximately 99 KDa (**Figure 1**). Regnase-3 emerged as a critical, non-redundant regulator that operates in immune cells such as myeloid, dendritic, and mast cells, where it modulates the type I interferon (IFN) response (63). It has been shown that the conservativeness between Regnase-1 and Regnase-3 of NYN/PIN domain and CCCH domain is 84.4% and 76% respectively (40).

Its transcription is induced by IFN signaling, forming an auto-regulatory negative feedback loop that tempers levels of *Ifnb1* and mRNAs encoding for interferon-stimulated genes (ISG) (64). Constitutive Regnase-3 KO mice develop hypertrophic lymph nodes and exhibit a systemic increase in IFN signaling, which disrupts germinal center formation and alters immune cell populations (65), confirming that Regnase-3 is an essential homeostatic protein (63).

Regnase-3 also plays a complex role in fine-tuning inflammatory responses by regulating key cytokines in a cell-type-specific manner. In mast cells, Regnase-3 physically interacts and cooperates with Regnase-1 to degrade *Tnf* mRNA upon cell activation. However, this interaction is part of a broader reciprocal feedback loop; indeed Regnase-3 destabilizes Regnase-1 transcripts, while Regnase-1 degrades Regnase-3 mRNA. This mutual regulation ensures a dynamic balance between Regnase-3 and Regnase-1 levels, allowing for precise control over shared target networks (e.g., *Il6* and *Immediate early response gene 3*, *Ier3*) (40). Simultaneously, each RNase protein maintains its unique target specificity: *Il12b* and v-rel avian reticuloendotheliosis viral oncogene homolog (*c-Rel*, a member of the NF−κB family of transcription factors) in the case of Regnase-1, while Regnase-3 uniquely governs interferon-related transcripts (66). However, Regnase-3 does not influence the expression of IL-1β in glioblastoma cells (40).

This regulatory role of Regnase-3 extends to the maintenance of tissue homeostasis, particularly in the skin. In keratinocytes, Regnase-3 is expressed and functions as a crucial brake on proliferation. It interacts with structural and signaling proteins like members of the 14-3–3 family and Keratin 14, forming complexes that influence cell cycle progression. Mechanistically, Regnase-3 expression peaks in peri-mitotic cells and is critical at the G2/M phase transition. Here, it helps to restrain the expression of key cell cycle regulators, including cyclin A2 (a regulator of S and G2 phases) and cyclin B1 (the master regulator of mitosis). The silencing of Regnase-3 leads to a significant accumulation of these cyclins in cells, causing dysregulated progression through the late cell cycle stages and thereby disrupting the delicate balance between epidermal growth and differentiation (67, 68).

Beyond the regulation of cell proliferation and immunity, Regnase-3 contributes to neural integrity. While its specific molecular functions in the CNS are still being elucidated, human genetic evidence strongly underscores its importance for long-term brain health. Genome-wide association studies identified ZC3H12C polymorphisms (rs4754425) as protective in late-onset Alzheimer’s disease, linking Regnase-3’s RNA regulatory function to neuronal maintenance and cognitive stability. Although this genetic association strongly suggests a role for Regnase-3 in brain health, whether polymorphisms alter its expression, stability, or catalytic activity remains to be experimentally undefined.

This finding positions Regnase-3 within a network of maternally imprinted genes, highly expressed in brain regions that are critical for cognition and memory, such as the cerebral cortex and hippocampus. The association implies that dysregulation of this post-transcriptional brake may disrupt the delicate cellular stability required to prevent a neurodegenerative pathology that emerges during aging. This observation, combined with the role of Regnase-3 in keratinocyte cycle control, indicates that Regnase-3’s regulatory reach extends beyond classical immunology to encompass fundamental processes of cell fate and tissue integrity (69).

### 3.4 Regnase-4 (ZC3H12D/MCPIP4): a broad regulator with links to oncogenesis

Regnase-4 is a protein of 527 aa with a molecular weight of approximately 58 KDa (**Figure 1**), and like Regnase-3 operates as a broad-spectrum post-transcriptional regulator, with targets that extend from classic inflammatory mediators to genes controlling cell cycle and proliferation. Peripheral B and T cells express Regnase-4 and upon anti-CD3 stimulation, they accumulate Regnase-4 at cytoplasmic GW-bodies, namely a functional subclass of P-bodies (70), which are hubs for mRNA decay (71) and storage (72). In human memory CD4+T cells the expression of Regnase-4 is under the regulation of Basic Helix-Loop-Helix Family Member E40 (BHLHE40) gene. The CRISPR-Cas9-mediated inactivation of e *ZC3H12D* in these cells enhances the expression levels of *TNF* and *IL22* mRNAs, suggesting that Regnase-4 contribute to the regulation of human memory T lymphocytes (73).

However, the ability of Regnase-4 to degrade mRNA transcripts is not limited to a single pathway; indeed Regnase-4 targets a diverse set of messengers including *Il6* and *Il1b*, the transcription factor *c-Fos*, and the cell-cycle regulator cyclin-dependent kinase inhibitor 1A (*Cdkn1a*). This wide target repertoire suggests a role that integrates inflammatory control with the regulation of cellular growth (28, 74).

*In vivo* evidence from the Regnase-4-deficient mice [in early studies Regnase-4 was reported with the name tumor suppressor gene transformed follicular lymphoma, TFL (75)] confirms its non-redundant role in immune regulation. Indeed, Regnase-4 deficiency does not appear to affect the early lymphopoiesis and KO mice develop normally and appear healthy under baseline conditions (72). However, they exhibit a heightened inflammatory response upon an immune challenge (72, 73, 76). The stimulation of splenic B cells obtained from these KO mice results in a strong upregulation of both IL-6 and IL-10 (72). For instance, in a model of LPS-induced acute lung injury, the loss of Regnase-4 results in exacerbated inflammation, characterized by elevated levels of NF-κB, IL-6, and TNF-α. These findings collectively underscore its fundamental role in tempering adaptive and innate immune responses (73, 77).

A distinctive feature of Regnase-4 is its capacity to form a heteromeric complex with Regnase-1; the two proteins co-localize in cytoplasmic GW-bodies. Despite this physical interaction, biochemical evidence indicates they act independently in degrading shared targets like *Il6* mRNA. This interaction may provide a multi-layered mechanism for fine-tuning the expression of critical shared transcripts (74, 78).

RNA sequencing of B cells obtained from CD20*^TAM^*^Cre^*Zc3h12a*^flox/flox^ mice in which Regnase-1 has been deleted displayed up-regulated levels of Regnase-4, suggesting a potential compensatory role of this member of the Regnase family in cells lacking Regnase-1 (50). Emerging evidence connects Regnase-4 dysregulation to oncogenic processes. Reduced expression levels of Regnase-4 have been reported in follicular lymphoma and lung adenocarcinoma, where loss of its mRNA-degrading function permits aberrant accumulation of cytokine and growth factor genes, suggesting that Regnase-4 operates tumor suppressive functions (75, 79).

## 4 Multi-layered regulation of Regnase proteins

The precise function of Regnase proteins in immunity is determined by a multi-layered regulatory network that operates at transcriptional, post-transcriptional, and functional levels. This intricate control ensures that their potent mRNA-decaying activity is activated in the correct cell type, at the right time, and in response to the appropriate signal. We outline some of these interactions from transcriptional induction to protein modification, highlighting how such regulation tailors Regnase activity to innate and adaptive immune contexts.

### 4.1 Mechanisms of transcriptional and post-transcriptional gene regulation

*Zc3h12* genes are subject to distinct transcriptional regulation programs that adapt their expression to specific immune contexts, forming the first layer of their functional diversification. *ZC3H12A* is a classic immediate-early response gene. Its promoter contains functional binding sites for the transcription factors NF-κB and Signal Transducer and Activator of Transcription (STAT)-3, both activated downstream receptors like TLR, IL-1R, and IL-6R (80). This mechanism ensures that the initiation of an inflammatory signal is accompanied by the activation of Regnase-1 synthesis. Such activation establishes a critical negative feedback loop; the newly synthesized Regnase-1 protein then acts to degrade the mRNAs encoding inflammatory mediators, such as *Il6*, thereby preparing the system for the timely resolution of the response (43).

Beside inflammatory mediators, in the duodenum Regnase-1 directly destabilizes the expression of transferrin receptor 1 (*TfR1*) and Egl-9 family hypoxia-inducible factor 3 (*PHD3*) mRNAs. In this organ, Regnase-1 expression is inducible and is under the regulation of hypoxia-response element that are activated by the hypoxia inducible factor 2a (HIF2a). Therefore, in absence of iron, Regnase-1 affects messengers of iron-controlling genes, promoting duodenal iron uptake (81).

*ZC3H12C* activation is independent of NF-κB, but Regnase-3 expression is primarily governed by the interferon regulatory factors IRF3 and IRF7. This aligns its induction perfectly with antiviral innate immunity and type I interferon signaling, positioning Regnase-3 as a specialized feedback regulator dedicated to constraining the interferon pathway (64). Both *ZC3H12B* and *ZC3H12D* are not subject to a fine transcriptional control, but they typically exhibit a more stable, constitutive, often tissue-specific, expression patterns. For instance, *ZC3H12B* shows high basal expression in the brain, and its transcription is not strongly induced by common immune signals. This observation suggests that both genes may have a primary role in maintaining basal RNA homeostasis, providing a first line of post-transcriptional control in specialized tissues, rather than serving as rapid-response elements, such as Regnase-1 and Regnase-3 (58, 74).

Post-translational modifications (PTMs) provide the most dynamic layer of control that fine-tunes Regnase’ s protein activities in response to signaling. The regulatory landscape modulating Regnase-1 serves as the prototype for the entire family of genes. As mentioned above, upon inflammatory activation, namely IL-1R, TLR, or T-cell receptor (TCR) signaling, Regnase-1 levels are increased in cells. However, the protein is phosphorylated by IKKβ/IRAK1, creating a docking site for the SCF^β-TrCP^ (Skp1-Cullin-F-box protein complex with the beta-transducin repeat-containing protein F-box protein) E3 ubiquitin ligase. This modification triggers K48-linked polyubiquitination and proteasomal degradation, temporarily lifting mRNA repression to allow a pulse of cytokine production (43). Conversely, the deubiquitinase Ubiquitin Specific Peptidase (USP)-10 removes these chains, stabilizing Regnase-1 and reinforcing its anti-inflammatory functions (82).

A complementary mechanism involves IRAK1-dependent phosphorylation, that promotes Regnase-1 binding to proteins that belong to the 14-3–3 family. This interaction is highly specific, primarily involving the isoforms 14-3-3β (YWHAB), 14-3-3γ (YWHAG), and 14-3-3ϵ (YWHAE). This association stabilizes protein levels, yet inhibits RNase activity of Regnase-1, thus functioning as a molecular pause mechanism that transiently stops mRNA decay while retaining Regnase-1 for subsequent use (41). The extent to which Regnase paralogs are controlled by analogous PTMs remains largely unknown. Given the conserved catalytic architecture shared across the family, divergent PTM profiles, including ubiquitination or paralog-specific protein interactions, may represent plausible mechanisms driving differences in stability and subcellular localization among Regnase-2, -3, and -4.

Notably, a recent study on paralog evolution shows that diversification of PTM patterns is a common mechanism by which structurally related proteins acquire non-redundant functions (83). Mapping these modification networks represents a major future direction for understanding Regnase functional diversification.

### 4.2 Regnase proteins in immunity

Within the innate immune system, it is generally conceived that Regnase-1 serves as a molecular timer that restrains excessive inflammation in tissues, while allowing effective host defense. In macrophages, Regnase-1 is a key regulator of cell polarization. Its depletion *via* lentiviral shRNA in models of liver ischemia/reperfusion injury (IRI) disrupts the M1/M2 balance, skewing macrophages to acquire a pro-inflammatory M1 cell phenotype (**Figure 2A**) (7, 84). This phenotype occurs through the dysregulation of key signaling pathways, including the above-mentioned NF-κB, but also the CCAAT/enhancer-binding protein beta (C/EBPβ), and the Peroxisome Proliferator-Activated Receptor (PPAR)-γ proteins (85), (**Figure 2A**). Similarly, Regnase-1 is a critical negative regulator of neuroinflammation in microglia. In these cells it is induced by High-Mobility Group Box (HMGB)-1, belonging to the damage-associated molecular pattern (DAMP) response. Once expressed, Regnase-1 degrades pro-inflammatory transcripts such as: *Il1b* and *Il6*, thereby limiting HMGB1-mediated neuronal toxicity. Knockdown of Regnase-1 in immortalized microglia increased proinflammatory cytokines expression, similarly the cell medium from Regnase-1 deficient microglia under HMGB1 treatment is more toxic to neurons than the medium obtained from experimental wildtype controls (**Figure 2B**) (43, 86).

In Regnase-1 knockout (KO) mice, the helper T (Th) cell population is increased, with elevated numbers of Th1, Th2, and Th17 cells detected (87). IL-17 signaling is exacerbated (84), supporting the idea that Regnase-1 functions as a physiological regulator that prevents excessive T-cell–mediated immune responses. This concept is further supported by evidence that, following TCR stimulation, Regnase-1 is cleaved at Arg111 by the paracaspase mucosa-associated lymphoid tissue lymphoma translocation protein 1 (MALT1), thereby enabling proper T-cell activation (84). This degradation is a prerequisite for clonal expansion of cells, as it releases brakes on targets molecules, such as *Il2* and allows for the robust expression of effector genes. This pathway appears peculiar of Regnase-1; indeed, it operates in a distinct manner from the related RNA-binding protein Roquin. While Roquin targets translationally inactive mRNAs in P-bodies, Regnase-1 specifically degrades translationally active mRNAs associated with ribosomes, allowing it to exert immediate control over the proteomic output of T-cell activation (7, 88).

Regnase-1 exerts a critical role in B cells as shown in a conditional knockout mouse line *Zc3h12a^flox/flox^*Mb1^Cre^ (89). KO mice displayed short survival, disrupted follicular architecture in the secondary lymphoid organs, hyperimmunoglobulinemia and in general high frequencies of abnormal B cell populations (50). The same team of authors generated a conditional KO mouse model using a hCD-20-driven Tamoxifen-dependent Cre allele (90). Inducible inactivation of Regnase-1 resulted in aberrant differentiation of B cells with an augmented germinal center (GC) and plasma cell response to immunization. Characterization of mRNA profiles in cKO B cells form these mice showed alterations of genes associated with survival, activation, and differentiation and genes that are induced upon BCR stimulation (50).

Regnase-1 exerts a more pleiotropic effect contributing to the self-renewal of hematopoietic stem and progenitor cells (HSPCs) by controlling the stability of GATA binding protein 2 (Gata2) and T-cell acute lymphocytic leukemia protein 1 (Tal1/Scl) mRNA (91).

Beyond regulating HSPC self-renewal, Regnase-1 and Regnase-3 also cooperate to enforce lymphoid lineage commitment by post-transcriptionally restricting myeloid differentiation pathways. In double-knockout HSPCs, loss of both Regnase-1 and Regnase-3 leads to severe lymphopenia—particularly of B-lineage cells—and a reciprocal expansion of myeloid progenitors. Mechanistically, they jointly degrade mRNAs encoding lineage-determining factors such as *Nfkbiz* (NF-kappa-B inhibitor zeta, IκBζ); unchecked *Nfkbiz* expression in HSPCs suppresses lymphopoiesis and promotes myeloid fate. This reveals a previously unappreciated, redundant role for Regnase-1 and Regnase-3 in licensing early lymphoid development by dampening pro-myeloid transcriptional networks (92).

A growing body of work now places Regnase family RNases as key post-transcriptional checkpoints in innate lymphoid cell (ILC) biology, with the clearest evidence in ILC2s. Regnase-1 acts as an intrinsic brake on type 2 responses: mice with ILC2-restricted or hematopoietic *Zc3h12a* deficiency develop spontaneous expansion and activation of lung ILC2s, upregulate profibrotic transcriptional programs (including *Egr1*), and exhibit aggravated bleomycin-induced pulmonary fibrosis. Low Regnase-1 expression in human ILC2s associates with increased circulating ILC2 numbers and worse idiopathic pulmonary fibrosis outcomes (93). Conversely, Regnase-1 must be degraded by the IKK complex to permit full IL-33- and IL-25-driven ILC2 proliferation and effector cytokine production; stabilization of Regnase-1 in *Zc3h12a*^AA/AA^ ILC2s – in mice where *Zc3h12a* alleles are mutated to encode Regnase-1 S435A/S439A that is resistant to IKK complex-mediated degradation— selectively destabilizes *Il2ra* and *Il1rl1* mRNAs, blunts type 2 cytokine output, attenuates acute allergic airway inflammation, and delays helminth expulsion (94). These findings position Regnase-1 within a broader landscape of RNA-binding proteins that shape innate immune sensing and cytokine networks at mucosal barriers (95), suggesting that Regnase-dependent decay of cytokine and receptor transcripts is an important, and still underexplored, layer of regulation for ILC-mediated tissue homeostasis, fibrosis, and allergic inflammation.

The role of Regnase-1 in infection is complex and context-dependent. Although it generally restrains inflammation, this function is not always beneficial to the host. In some bacterial infections, its inhibition can enhance immune responses and improve pathogen clearance, highlighting the delicate balance between immune regulation and effective host defense.

In a model of *Klebsiella pneumoniae* (KP) pneumonia obtained by oropharyngeal-mediated infection of mice, survival and weight loss were significantly ameliorated in Regnase-1 heterozygous mice (96). This protection does not appear to be mediated by classic NF-κB related cytokines but through an enhanced type I IFN (IFN-β) expression, and such protection derives from hematopoietic and nonhematopoietic cells. Invitro the half-life of *Irf7* mRNA levels was considerably increased in the presence of Regnase-1 haploinsufficiency, causing the activation of IFN-β transcription. Increased levels of IFN-β obtained in these mice can confer an anti-inflammatory advantage that is sufficient to prolong their survival upon KP infection (96). Among nonhematopoietic cells, Regnase-1 is highly expressed by alveolar type 2 cells (AT2) that are also involved in KP infection. However, the conditional deletion of *Zc3h12a* in AT2 cells did not enhance survival when compared with controls, suggesting that another group of nonhematopoietic cells that express high levels of Regnase-1 may contribute to KB resistance (97).

Beyond Regnase-1, other members of the *Zc3h12* gene family provide specialized layers of immune control. Regnase-3, for instance, is transcriptionally uncoupled from NF-κB and is induced by IRF3 and IRF7, positioning it as a dedicated feedback regulator of the type I IFN pathway itself. This specialization ensures that distinct arms of the innate immune response are independently controlled by different Regnase proteins (63, 64).

The Regnase proteins family exerts profound and multi-faceted control over adaptive immunity, with Regnase-1 acting as a central gatekeeper of T-cell activation and differentiation. In naive T cells, Regnase-1 maintains a state of cell quiescence by constitutively degrading a suite of mRNAs that encode for critical activation molecules, including the costimulatory receptors *Inducible T-cell COStimulator* (*Icos*), *Tumor Necrosis Factor Receptor Superfamily Member 4* (*Tnfrsf4*, commonly known as OX40) and the NF-κB subunit *c-Rel*. As stated above, the repression of Regnase-1 is so critical for the maintenance of T cell homeostasis that CD4^+^ T cell–specific deletion of Regnase-1 provokes fulminant autoimmunity (**Figure 2C**) (7, 84). Mutant mice accumulate activated effector/memory T cells and plasma cells, develop hypergammaglobulinemia, and rapidly succumb to fatal systemic inflammation. These results underscore Regnase-1’s essential role as a T cell–intrinsic immune checkpoint (7, 84).

Beyond initial activation of T cells, Regnase-1 fine-tunes the T-helper cell fate acquisition. By regulating the stability of mRNAs such as the Il-6 receptor (Il-6r) and the Il-2 receptor/CD25, it can influence the sensitivity of T cells to polarizing cytokines, thereby shaping the balance between pro-inflammatory and regulatory responses (43).

Regnase-4 has additionally been identified as a major modulator of human memory T lymphocytes. Inflammatory cytokine-producing human memory T cells exhibit constitutive NF-κB activation, which is sustained in part by the suppression of Regnase-4 (73, 98, 99). BHLHE40 attenuates *ZC3H12D* expression, thereby releasing a brake on inflammatory transcripts and reinforcing the pro-inflammatory phenotype of cells. This observation positions Regnase-4 as a crucial RNase in defining the functional state of human memory T cells (**Figure 2D**) (73). This critical role of Regnase-4 in restraining inflammation is supported by *in vivo* observations. Constitutive Regnase-4 KO mice exhibit hyperproliferation of activated T and B cells and excessive secretion of IL-2, IL-6, and IL-17A (**Figure 2D**). Messenger RNA stability assays confirmed that Regnase-4 directly targets the 3′ UTRs of transcripts encoding these cytokine (72). Experimental autoimmune encephalomyelitis (EAE), the classical murine model of multiple sclerosis (100), further highlights the immunoregulatory function of Regnase-4. Regnase-4–deficient mice exhibit markedly exacerbated disease, developing more severe paralysis along with a pronounced accumulation of pathogenic Th17 cells infiltrating the CNS during the chronic phase of inflammation (72). Interestingly, other Regnase paralogs undergo dynamic regulation during neuroinflammatory conditions.

## 5 When regulation goes rogue: the pathological power of the Regnase gene family

The biological significance of the Regnase proteins becomes most evident in pathological conditions, when the precision of their post-transcriptional regulatory machinery is somehow disrupted. Indeed, under physiological conditions, Regnase proteins operate as molecular sentinels that ensure the transient expression of cytokines, signaling mediators, and TFs that are required for immune activation and resolution (101). Through their coordinated RNA-degrading activity, Regnase proteins preserve tissue integrity and prevent excessive or prolonged inflammation. However, perturbations in this finely balanced system, whether due to genetic mutations, post-translational inactivation, or sustained inflammatory signaling, can fundamentally alter immune and tissue homeostasis. Evidence from both human genetics and animal models demonstrates that each Regnase paralog (Regnase-1 to -4) contributes to the maintenance of tissue-specific immune equilibrium. Regnase-1 and Regnase-3 are indispensable for limiting cytokine-driven inflammation in epithelial and myeloid compartments; Regnase-2 modulates cell proliferation and stress responses in neural and mucosal tissues; and Regnase-4 acts as a late-phase suppressor of lymphocyte activation. Together, they form a distributed post-transcriptional control network that links RNA metabolism to immune and structural homeostasis across organ systems (**Table 1**).

**Table 1:** Overview of pathological conditions linked to Regnase genes.

Pathology	Regnases involved	Bioprocess modulated	Reference
Inflammatory Skin Disease/Atopic Dermatitis	Regnase-1	Anti-inflammatory brake in skin; control of local cytokine milieu (e.g., Th2 cytokines, IgE, chemokines Ccl2, Ccl7, Ccl8, Cxcl1)	(106)
Inflammatory Skin Disease/Psoriasis	Regnase-1	Keratinocyte homeostasis; repression of IL-36, S100a8/a9, Sprr genes; restraint of IL-17–driven inflammation	(87, 107)
	Regnase-3	Pathogenic amplifier; reciprocal regulation with Regnase-1; enhancement of IL-17/IL-36 inflammatory programs; regulation of keratinocyte proliferation/differentiation	(40)
Cardiovascular Disease	Regnase-1	Myocardial Infarction: Cardiomyocyte repair; suppression of inflammation and P53/ferroptosis pathway; promotion of reparative M2 macrophages	(110)
	Regnase-1	Atrial Fibrillation: Cardiac fibrosis and remodeling (pathogenic role via miR-26a-5p/FRAT1/Wnt pathway)	(111)
	Regnase-1	Marfan Syndrome: Vascular inflammation control; reduction of pro-inflammatory cytokines and matrix metalloprotease (MMP) expression	(113)
Pulmonary Disease	Regnase-1	Pulmonary Arterial Hypertension (PAH): Pulmonary vascular remodeling; restraint of inflammation in macrophages	(115, 116)
	Regnase-1	Pulmonary Injury/Fibrosis: Resolution of acute respiratory inflammation and chronic fibrotic remodeling	(117)
Inflammatory Bowel Disease (IBD)/Colitis	Regnase-1	Intestinal epithelial cell (IEC) metabolism; mTOR and purine synthesis pathway regulation; mucosal regeneration	(118)
	Regnase-1	Monocyte-to-macrophage maturation in mucosa (via ATF3-AP1S2 axis)	(119)
Renal Homeostasis/AKI & CKD	Regnase-1	Macrophage-mediated renal inflammation; degradation of Irf4 mRNA to limit pro-inflammatory cytokines	(123)
	Regnase-3	Macrophage polarization; degradation of pre-mRNAs for M1-polarizing genes	(150)
Cancer	Regnase-1	Colorectal Cancer: Suppression of IL-17 signaling via degradation of NFKBIZ (IκBζ) mRNA	(132)
	Regnase-1	Clear Cell Renal Cell Carcinoma: Suppression of oncogenic HIF2α (EPAS1) signaling	(136)
	Regnase-1	Pancreatic Cancer: Suppression of MYCN oncogene	(129)
	Regnase-2	Glioblastoma: Cell cycle regulation; degradation of mRNAs for cell cycle regulators (CCND1, CCNE1, AURKA, PLK1)	(62)
	Regnase-3	Hyperplastic Skin Conditions: Keratinocyte proliferation and differentiation; suppression of cell cycle genes	(67, 104)
	Regnase-3	Vascular Inflammation: Suppression of TNFα-induced adhesion molecules (e.g., VCAM1) in endothelial cells	(66)
	Regnase-4	Lymphoma/Lung Cancer: Putative tumor suppression in lymphoid cells	(75, 79, 151)

A case report study shows a patient with private homozygous protein-truncating mutation in ZC3H12A leading to deficiency of Regnase-1 functionality. Soon after birth, this patient developed splenomegaly, autoimmune anemia and thrombocytopenia and later developed autoimmune hepatitis (102). The immunological assessment of bulk T cells revealed that γδ T cells expressing VCAM-1 and IFNγ genes were expanded, causing an aberrant interaction with B cells that in turn led to to systemic autoimmunity (102).

Effects deriving from Regnase gene inactivation have been widely studied in animal models. These experiments were done using conditional KO, obtained crossing *Zc3h12a^flox/flox^* mice with several transgenic mouse lines carrying tissue- or cell-specific Cre recombinases. For example, *LysM^Cre^* -mediated inactivation of Regnase-1 allowed the study of this member of the Regnase family in myeloid lineage cells, such as macrophages and neutrophils (49). *LysM*^Cre^*Zc3h12a*^flox/flox^ mice spontaneously develop a severe, age-dependent autoimmune phenotype that closely mirrors human systemic lupus erythematosus (SLE). Disease characterization revealed splenomegaly and lymphadenopathy, driven by a massive expansion of immune cells as shown by flow cytometry. Serological analysis, via ELISA assessment, confirmed the presence of classic SLE autoantibodies, including anti-double-stranded DNA (anti-dsDNA). Histological assessment of kidneys showed the presence of severe glomerulonephritis, with immune complex deposition and structural damage (49).

Transitioning from systemic manifestations to tissue-specific inflammation, psoriasis—a chronic IL-17–driven inflammatory skin disorder— and atopic dermatitis (AD)—a type 2 immunity–driven inflammatory skin disorder (103)— exemplify how Regnase proteins orchestrate immune responses in a distinct pathological setting. Moreover, it serves as a paradigmatic example of how different Regnase family members can exert opposing functions in the same microenvironment. In this condition, Regnase-1 functions as a key anti-inflammatory brake, while Regnase-3 acts as a pathogenic amplifier, establishing a post-transcriptional equilibrium that determines whether inflammation resolves or persists (104, 105).

In atopic dermatitis, Regnase-1 levels are decreased in the non-lesional and, overall, in lesional skins biopsies of patients (106). The induction of a model of atopic dermatitis (dust mite extract HDM/DNCB) in *Zc3h12a*^^+^/^−^^ mice resulted in worsening the phenotype with epidermal hyperplasia, edema higher numbers of inflammatory cells and increased serum IgE and Th2 cytokines (IL-4 and IL-5), compared with wild-type controls. Of note, a recombinant Regnase-1 protein administered in mice with atopic dermatitis symptoms alleviated the skin inflammation (106). The transcriptomic profiling (bulk RNA-seq) of full-thickness skin from *Zc3h12a*^+/-^ mice under atopic dermatitis conditions shows an overexpression of several chemokines, particularly *CC chemokine ligand (Ccl)2*, *Ccl3*, *Ccl8* (monocyte/macrophage chemoattractant), and *CXC chemokine ligand (Cxcl)1*, *Cxcl2*, *Cxcl12*, *Cxcl13* (neutrophil chemoattractant) when compare compared with control mice (106).

Regnase-1 functions within keratinocytes, the primary epithelial cells of the skin, to control the local cytokine milieu and maintain cutaneous immune homeostasis. This was demonstrated using *Zc3h12a^flox/flox^* mice crossed with *Krt14^Cre^* transgenic mice (in this study referred to as Mcpip1^EKO^) to obtain a specific deletion of Regnase-1 in keratinocytes (107). Starting from the age of 4 months, *Krt14^Cre^*/*Zc3h12a^flox/flox^* mice spontaneously develop an inflammatory skin phenotype with wounds around their cheeks, ears, necks, and trunks. The upregulation of genes related to inflammation and keratinocyte differentiation, including *Il36a*, *Il36g*, the calcium-binding protein *S100a8* and *S100a9*, and the *Small proline-rich proteins (Sprr)2d/2e/2h*, as well as pro-inflammatory cytokines such as *Tnf*, *Il6*, *Il18*, and *Il33*. With aging, *Krt14^Cre^*/*Zc3h12a^flox/flox^* mice progressively worsened this phenotype (**Figure 2E**) (107).

Regnase-1 was elevated ~10-fold in human psoriasis lesions compared to non-lesional skin from the same patient or to skin from healthy controls. Similarly, the application of imiquimod (IMQ) to the skin of mice upregulated the expression of *Zc3h12a* mRNA (87). Of note, in the IMQ-induced model of psoriasis with a robust skin inflammatory response that recapitulates key histological and molecular hallmarks of the human disease, including epidermal hyperplasia (acanthosis), parakeratosis, and dense infiltration of neutrophils and lymphocytes; topical application of a TLR7/8 agonist triggers a robust psoriasiform inflammation characterized by epidermal hyperplasia, scaling, and immune infiltration (89). The up regulation of Regnase-1 is interpreted as a compensatory and protective response. Indeed, Zc3h12a^+/−^ mice receiving the IMQ treatment showed increased disease severity when compared to their controls (87). However, in the absence of one Regnase-1 allele, levels of *Il17a*, *Il17f* and *Il17c* mRNA where not altered when compared to controls, nor were *Il23* and its receptor *Il23r* were upregulated (87). The effect of Regnase-1 haploinsufficiency was probably due to enhanced downstream IL-17R signaling (87).

In other models, involving keratinocyte-specific deletion (*Krt14^Cre^*/*Zc3h12a^flox/flox^* or *K5*^Cre^/*Zc3h12a^flox/flox^*), or *Zc3h12a*^+/−^ mice the loss of Regnase-1 consistently exacerbates, and in some cases precipitates, psoriasis-like dermatitis (**Figure 2E**) (87, 107, 108). The worsened inflammation in Regnase-1–deficient mice model demonstrates that Regnase-1 is functionally necessary to restrain inflammation: when it’s absent or insufficient, the inflammatory responses (neutrophil infiltration, cytokine-induced genes, epidermal pathology) get amplified.

The level of Regnase-3 mRNA is slightly, but significantly, increased in psoriasis-derived patient skin tissues and in an experimental model of psoriasis, suggesting that Regnase-3 might participate to the disease pathobiology, possibly, through degradation of inflammatory mediators (40). In mice treated with IMQ, Regnase-1 was strongly upregulated showing a peak of expression on 3 days from stimulation. Regnase-3 showed similar kinetic but at much lower levels compared with Regnase-1. However, a potential regulatory loop between Regnase-3 and Regnase-1 exists, as Regnase-3 levels triplicated in the contest of keratinocyte Regnase-1 deficiency (40).

The identification of a single-nucleotide polymorphism (SNP) in human, located upstream of the transcriptional starting site of ZC3H12C and highly associated with psoriasis provides a possible explanation to these observations (109). This SNP increases the expression levels of Regnase-3 *in vitro* and in the same study, authors further confirmed that Regnase-3 transcripts are slightly upregulated in lesional skin biopsies of patients with psoriasis (68). It should be noted, however, that the functional impact of this polymorphism on Regnase-3 RNase activity— as opposed to its effect on transcript abundance—remains undefined, leaving open whether enhanced catalytic efficiency or altered target selectivity underlies its disease association. Using a combination of conditional Regnase-3 KO models, authors of this study demonstrated that Regnase-3 is positively associated with psoriasis pathogenesis, and highly expressed by macrophages and plasmacytoid dendritic cells possibly operating in the early phase of immune cell activation (68).

Together, these findings delineate a cell-type–specific, bidirectional regulatory circuit in which Regnase-1 and Regnase-3 reciprocally modulate inflammatory gene expression, balancing protective feedback and pathogenic amplification. The outcome of this interaction determines whether psoriatic inflammation is resolved or evolves into chronic disease.

### 5.1 Regnase-1 in cardiac and pulmonary homeostasis and stress response

Regnase-1 deficiency or dysregulation contributes to heart failure, atrial fibrillation, impaired myocardial infarction (MI) recovery, and pulmonary hypertension. The role of Regnase-1 after myocardial infarction was investigated using cardiomyocyte-specific Regnase-1 knockout KO mice (*αMHC^Cre^*/*Zc3h12a^flox/flox^*) that were subjected to permanent ligation of the left anterior descending coronary artery. The loss of Regnase-1 leads to increased inflammation, larger infarct size, lower survival, and impaired cardiac repair. Persistent elevation of *Il6* mRNA was observed in cKO mice as well as high numbers of infiltrating CD45^+^, CD68^+^, and CD3^+^ cells, suggesting that Regnase-1 protects the heart against hemodynamic stress, (**Figure 2F**) (110).

However, the function of Regnase-1 is highly context-dependent and can vary significantly across different cardiac cell types. Paradoxically, the role of Regnase-1 in the atrium appears to be the opposite. In a rat model of atrial fibrillation (AF) induced by transaortic constriction, the cardiac overexpression of Regnase-1—by AAV9-mediated gene delivery—exacerbated atrial fibrosis and AF susceptibility. Further *in vitro* analysis, using H9C2 cells, an immortalized rat heart-derived cell line, demonstrated that Regnase-1 mediates this effect via the miR-26a-5p/FRAT1/Wnt signaling pathway (111).

Regnase-1 exhibits a more uniform protective function in vascular tissues. Overexpression of Regnase-1 in vascular tissue reduces inflammation, matrix degradation, and aortic dilation in an induced model of AF In a murine model of Marfan syndrome (112), intravenous delivery of a vascular-targeted AAV vector encoding Regnase-1 efficiently transduced the aortic wall and induced robust Regnase-1 overexpression, leading to reduced proinflammatory cytokines, decreased matrix metalloprotease (MMP) expression and activity, improved elastin architecture, and smaller aortic diameters. These results support AAV-mediated Regnase-1 overexpression as a potential gene therapy strategy to inhibit aortic aneurysm progression (113). Regnase-1 overexpression resulted in a marked decrease in inflammatory transcripts, such as *Il6*, *Il12*, and *Ccl2* in aortic tissue (113). The critical importance of Regnase-1 in tissue homeostasis is also evident from observations made on developing embryos, where its levels must be precisely regulated. Overexpression of Regnase-1 during embryogenesis disrupts heart development, indicating that precise control of Regnase-1 is necessary for normal cardiac formation (55).

The pulmonary vasculature represents another critical site where Regnase-1 restrains inflammation-driven remodeling. Pulmonary arterial hypertension (PAH)—a progressive disorder characterized by increased pulmonary vascular resistance, right ventricular hypertrophy, and ultimately heart failure (114)—has been directly linked to *ZC3H12A* loss-of-function variants. Whole-genome sequence of patients with PAH, available in the National Cerebral and Cardiovascular Center (NCVC) Biobank repository, screened for *ZC3H12A* mutation allowed the identification of a missense mutation (D426G) that compromises Regnase-1 protein stability (115).

Hemodynamic measurements of both human PAH patients and *LysM^Cre^/Zc3h12a^foxl/flox^* conditional KO mice revealed elevated right ventricular systolic pressure (RVSP), which is a pathological hallmark of PAH. Real time PCR analysis of alveolar macrophages isolated from PAH patients, showed a significant reduction of *ZC3H12A* mRNA levels when compared to levels established in healthy controls. Immunohistochemistry of lung tissue biopsies from these patients demonstrates marked perivascular infiltration of inflammatory macrophages, mirroring findings observed in *LysM^Cre^/Zc3h12a^foxl/flox^* mice, which spontaneously develop a PAH-like pathology (116).

The expression of Regnase1 is negatively regulated by Regnase-1–mediated mRNA degradation via the stem-loop structure that are placed in its own 3′UTR (43). Using antisense phosphorodiamidate morpholino oligonucleotides (MOs) to reduce the protein’s binding to the 3’UTR is a feasible strategy to increase Regnase-1 levels. Intratracheally administration of these MOs reduced acute respiratory inflammation and chronic fibrosis acute and chronic inflammatory in these disease models, indicating that Regnase-1 is a valuable target for therapeutic interventions (117).

### 5.2 Gut-related diseases

Genetic and experimental evidence strongly establishes Regnase-1 as a central regulator of intestinal immune homeostasis. Regnase-1 coordinates the balance between tolerance and inflammation in the gut, integrating immune, epithelial, and metabolic pathways. When this post-transcriptional control fails, the result is uncontrolled cytokine signaling, impaired barrier function, and chronic intestinal inflammation.

In mice with an intestinal epithelial-specific deletion of Regnase-1 (*Vil^Cre^/Zc3h12a^flox/flox^*, in this study referred to as Regnase-1^ΔIEC^), mucosal regeneration after dextran sulfate sodium (DSS)-induced colitis is profoundly disrupted. Combining RNA Immuno Precipitation (RIP)-seq and metabolomic analysis, it was observed that Regnase-1 normally acts as a metabolic brake, driving the degradation of mRNAs encoding components of the mTOR pathway and purine synthesis (118). The loss of Regnase-1 leads to metabolic overdrive and dysfunctional epithelial repair, both independent of its classic role in cytokine regulation (118).

Regnase-1 also directs the fundamental cellular process of monocyte-to-macrophage maturation, which is involved in the maintenance of mucosal integrity. *LysM^Cre^/Zc3h12a^flox/flox^* mice challenged with DSS to induce colitis exhibited exacerbated inflammation of the gut mucosa. Flow cytometric and single-cell RNA-seq analyses revealed a specific impairment in monocyte-to-macrophage maturation, with an accumulation of inflammatory monocytes and a deficit in mature, homeostatic macrophages. This defect was driven by the Activating Transcription Factor (ATF)-3/Adaptor Protein Complex 1, Sigma 2 Subunit (AP1S2) axis, wherein Regnase-1 deficiency stabilizes *Atf3* mRNA, leading to ATF3-mediated repression of *Ap1s2*, a gene essential for the functional maturation of intestinal macrophages. The same study shows that the protein expression of Regnase-1, ATF3 and AP1S2 are elevated in the colonic mucosa from patients with active inflammatory bowel disease, highlighting the critical role of Regnase-1-ATF3-AP1S2 in inflammatory disease of the gastrointestinal tract (119).

### 5.3 Renal homeostasis and kidney injury

The kidney represents one of the most tightly regulated interfaces between inflammation and tissue repair (120), and members of Regnase family play a pivotal role in balancing these processes. Both Regnase-1 and Regnase-3 have emerged as crucial post-transcriptional checkpoints that determine whether an acute injury resolves or progresses toward a chronic kidney disease (CKD) condition (121). Their coordinated activity in immune and parenchymal cells ensures that the inflammatory phase following tissue damage remains self-limiting and that regenerative pathways are appropriately engaged. Regnase-1 was identified as a molecular sentinel of immune homeostasis in models of acute kidney injury (AKI) and IRI (122), two conditions characterized by transient hypoxia followed by reperfusion-induced sterile inflammation. The loss of Regnase-1 in macrophages of *LysM^Cre^/Zc3h12a^flox/flox^* mice exacerbates inflammation following IRI. Histological examination of kidneys reveals pronounced tubular necrosis, extensive interstitial infiltration of CD68^+^ macrophages and Ly6G^+^ neutrophils, and elevated levels of serum creatinine, indicating renal dysfunction (123). Both RIP and reporter assays demonstrate that Regnase-1 directly binds and degrades *Irf4* mRNA in macrophages. IRF4 normally drives the transcription of pro-inflammatory cytokines and chemokines that perpetuate kidney inflammation and promote chronic disease progression (124). Consequently, in the absence of Regnase-1, *Irf4* mRNA is stabilized, leading to an accumulation of IRF4^+^ macrophages within the renal interstitium. Flow cytometry assessment of macrophages confirms that these cells produce excessive IL-6, TNF-α, and CCL2, thus amplifying local inflammation and promoting secondary tubular damage (123).

While Regnase-1 is a key sentinel in renal immunity, its homolog Regnase-3 provides a distinct layer of control that converges on the inflammatory polarization of kidneys -resident macrophages. In a model of AKI, macrophage-specific deletion of Regnase-3 using *LysM^Cre^/Zc3h12c^flox/flox^* mice, exacerbates acute injury and promotes the transition to chronic kidney disease (CKD). This detrimental outcome was driven by uncontrolled polarization of macrophages towards a pro-inflammatory M1 cell phenotype. RIP assay revealed that Regnase-3 exerts this regulation by binding and degrading primary transcripts (pre-mRNAs) of specific genes that orchestrate M1 macrophages polarization. Thus Regnase-3 acts as a critical upstream regulator that restrains macrophage-driven inflammation to preserve renal integrity after injury. However, Regnase-3 exerts different effects depending on the cell lineage in which it operates (125). To highlight these differences, two different mouse models were used to get inducible deletion of Regnase-3 in kidney epithelial cells; indeed, a *Pax8-*rtTA; *Tet*O^Cre^; *Zc3h12c^flox/flox^* model was used to get Regnase-3 inactivation in these cells. In this model, the *Pax8* promoter drives the reverse tetracycline-controlled transactivator (rtTA) specifically in renal tubular cells. Upon administration of doxycycline, the rtTA activates the TetO-Cre transgene, leading to Cre-mediated excision of the floxed Regnase-3 allele. To achieve an alternative deletion of Regnase-3 in myeloid cells, a *Rank^Cre^/Zc3h12c^flox/flox^* mouse model was also utilized. The Receptor Activator of Nuclear Factor Kappa-B (Rank) promoter ensures that Cre recombinase is expressed constitutively in resident kidney macrophages. This results in a cell-specific knockout of Regnase-3 within this innate immune cell population (125). When both mouse models were subjected to renal IRI, *Rank^Cre^/Zc3h12c^flox/flox^* mice showed an exacerbation of kidney injury by increasing macrophage recruitment, whereas the *Pax8-*rtTA; *Tet*O^Cre^; *Zc3h12c^flox/flox^* mice displays an improvement in kidney injury through its effects on cell death and wound healing capability of tubular epithelial cells. Therefore, the role of Regnase-3 in kidney injury depends on the cell type involved in the damage, which ultimately determines the detrimental or beneficial impact on kidney injury (125).

### 5.4 The immunological relationship between Regnase family members and cancer

The intersection between RNA metabolism and oncogenesis is a rapidly evolving field (126), and the Regnase family has emerged as a critical layer of post-transcriptional control linking immune regulation to cancer biology (127). These proteins contribute to the fine-tuning of mRNAs stability of genes encoding cytokines, transcription factors, and signaling molecules that orchestrate tumor-promoting inflammation, cell proliferation/apoptosis, angiogenesis, and immune evasion. Dysregulation by whether via mutation, epigenetic silencing, or post-translational degradation, can shift this balance, transforming protective immune mechanisms into tumor-supportive pathways. While Regnase-1 remains the best-characterized member of the family in cancer, growing evidence implicates Regnase-2, -3, and -4 as important modulators of oncogenic signaling and the tumor immune microenvironment (**Table 1**).

An interesting observation emerged from the analysis of patients with resectable pancreatic cancers who underwent surgery and whose tumor biopsies were evaluated for Regnase-1 expression levels. Patients with low Regnase-1 expression levels in the tumor were featured by significant shorter recurrence-free survival and overall survival than patients with high Regnase-1 expression levels. Interestingly, mice in which a pancreas-specific Regnase-1 KO has been used to develop an inducible focal pancreatitis revealed that a reduction of Regnase-1 levels fosters pancreatic cancers by myeloid-derived suppressor cell (MDSCs)-mediated evasion of antitumor immunity (128). In pancreatic cancer, Regnase-1 suppresses the proto-oncogene *MYCN*, and therefore a loss of Regnase-1 activity enhances the proliferative signaling in these cells (129). Parallel observations suggest that Regnase-1 plays a crucial role in shaping the tumor microenvironment (TME). Indeed, also in myeloid-specific KO models, the loss of Regnase-1 leads to an accumulation of MDSCs creating an immunosuppressive TME, impairing T cell activation and accelerating tumor progression (130). This highlights a dual function: intrinsic tumor suppression within malignant cells, and extrinsic modulation of anti-tumor immunity through myeloid regulation.

Patients with ulcerative colitis (131), a common form of inflammatory bowel disease associated with an increased risk of cancer, display mutation in ZC3H12A (47). Mice lacking Regnase-1 in intestinal epithelia develop tumors in the intestine. Differential gene expression analysis revealed that *Nfkbiz (IκBζ)* mRNA and its downstream genes are upregulated (132). Therefore, loss of Regnase-1 in tumor cells amplifies IL-17–driven inflammation, accelerating tumor growth, promoting epithelial proliferation (132).

Clear cell renal cell carcinoma (ccRCC), is the most common urological malignancies worldwide (133). During tumor progression the reduction of Regnase-1 correlates with renal cancer progression and better tumor vascularity. Indeed, the lack of Regnase-1 activity in grade III-IV cancers promotes the release of proangiogenic factors, acquisition of the mesenchymal phenotype, and the metastatic spread of ccRCC cells (134, 135). The overexpression of Regnase-1 in ccRCC cell lines increased cysteine-rich protein with Kazal motifs (RECK) levels, which are positively associated with better patient survival (136). Xeno transplanted NOD-SCID mice with cancer cells overexpressing Regnase-1 elevated the expression levels of the tumor suppressors TIMP3, RECK and PTEN, such results suggest that Regnase-1 can be used as valuable target for tumor suppression (136).

Recent findings reveal that Regnase-1 acts as a post-transcriptional checkpoint for effector lymphocytes. In natural killer (NK) cells, it directly targets *POU class 2 homeobox 2* (*POU2F2* also known as *OCT2)* and *NFKBIZ (IκBζ)* transcripts—both of which regulate IFN-γ production. Deletion of Regnase-1 enhances IFN-γ output and boosts cytotoxic activity, suggesting the existence of a finely tuned balance between immune activation and exhaustion (137).

Closely linked to cell proliferation, apoptosis is a form of programmed cell death that is frequently suppressed in cancer cells (138). Bcl-2 and Bcl-xL (Bcl-2 family member) are anti-apoptotic proteins which prevent the release of cytochrome c from mitochondria (139). Regnase-1 destabilizes Bcl-xL and Bcl-2-related protein A1, by physically interacting with their 3’UTR (140). The upregulation of Bcl-2 family members in a variety of tumors is part of a broad mechanism by which cancer cells develop resistance to apoptosis (141). Similarly, the expression of Birc3, which belongs to the family of inhibitors of apoptosis protein (IAP), is under the regulation of Regnase-1 (140). In breast cancer, the levels of Regnase-1 are downregulated, allowing cancer cells to increase levels of these anti apoptotic genes and therefore to skip apoptosis. Conversely, the over expression of Regnase-1 in breast cancer cells increased cell death (140).

Osteosarcoma is the most common primary malignancy of the bone affecting children and adolescents (142). In human tumor tissues, the expression levels of microRNA (miR)-421 are strongly upregulated and patients with high expression of miR-421 had a shorter overall survival than those with low expression of miR-421. Regnase-1 shows an inverse expression pattern, being significantly downregulated in tumor specimens characterized by elevated miR-421 expression. A luciferase reporter assay demonstrated that miR-421 directly binds to the 3′UTR of Regnase-1, contributing to its downregulation. The interplay between miR-421 and Regnase-1 contributes to the regulation of proliferation, invasion, and migration of osteosarcoma cells (143).

Altogether, this large body of experimental evidence collectively positions Regnase-1 as a multifaceted tumor suppressor, operating both within cancer cells and across the immune landscape to restrain inflammation, angiogenesis, and immune suppression.

While less investigated in this field, Regnase-2 plays an emerging anti-proliferative role that has been observed in the CNS. Glioblastoma multiforme (GBM) is one of the most aggressive malignancies of the CNS (144). In GBM samples Regnase-2 is significantly reduced reaching the lowest levels in n patients with high-grade GBM, and such low levels well correlate with the poor survival of these patients (61). The over expression of Regnase-2 in GBM cell lines directly degrades mRNAs encoding critical cell cycle regulators, including *Cyclin D1 (CCND1), Cyclin E1 (CCNE1), Aurora Kinase A (AURKA) and Polo-Like Kinase 1 (PLK1)*, leading to G1-phase arrest. Overexpression of wild-type, but not a catalytically inactive form of Regnase-2, suppresses proliferation and colony formation in these GBM cell lines, establishing Regnase-2 as a cell-intrinsic tumor suppressor that acts independently of cytokine regulation. These results position Regnase-2 as a molecular brake working on uncontrolled proliferation, and its loss may contribute to tumorigenesis in neural cancer cells through deregulation of the cell cycle machinery (62).

The roles of Regnase-3 in cancer are largely unknown. However, like Regnase-1 and -2 the expression levels of *ZC3H12C* in colorectal cancer specimens are significantly reduced. Moreover, the over expression of Regnase-3 in cell models affects colorectal cancer cell migration, therefore it can operate as a potential tumor suppressor gene (145). In keratinocyte-specific *Zc3h12c* KO (*Krt14^Cre^/Zc3h12c^flox/flox^* mice) loss of Regnase-3 results in epidermal hyperproliferation, aberrant differentiation, and upregulation of cell cycle–related genes, all of which are characteristic of hyperplastic precancerous development (104). Complementary to these observations, *in vitro* silencing of Regnase-3 increases keratinocyte viability and proliferation rates (67).

In vascular endothelial cells, the overexpression of ZC3H12C suppresses TNFα-induced expression of adhesion molecules (*Vascular Cell Adhesion Molecule 1*, *VCAM1*; *E-selectin*; *MCP-1*), reducing leukocyte adhesion and vascular inflammation (66). Collectively, these findings highlight Regnase-3 as a gatekeeper of tissue inflammation that indirectly suppresses inflammation-associated tumorigenesis by limiting the activity of TNF-α and the IL-17 pathway.

Regnase-4, originally identified as a putative tumor suppressor in follicular lymphoma and lung cancer, shares functional similarities with Regnase-1, but operates primarily in lymphoid cells. Loss-of-function mutations or chromosomal rearrangements affecting *ZC3H12D* have been reported in transformed follicular lymphoma [t(6;1)(q25;q21)] and sporadic lung cancer (75, 79).

### 5.5 Targeting Regnase: a new frontier in cancer and inflammation therapy

Members of the Regnase family have emerged as promising, manipulable checkpoint hubs with significant therapeutic potential in inflammatory diseases and cancer. Strategies are bifurcating into those that aim at increasing Regnase functionality to suppress harmful inflammation and those that aim at inhibiting Regnase activity to potentiate anti-tumor immunity. The first therapeutic strategy has been developed to boost Regnase-1 activity and can be used for treating autoimmune, fibrotic, and vascular diseases. A compelling approach utilizes antisense oligonucleotides (ASOs), specifically phosphorodiamidate MOs that are designed to target the self-regulatory stem-loop in the *ZC3H12A* 3’UTR (a mechanism detailed in earlier sections on post-transcriptional control). By blocking this auto-regulatory site, MOs prevent Regnase-1 from degrading its own mRNA, thereby stabilizing the transcript and increasing endogenous protein levels. This strategy has demonstrated efficacy in multiple preclinical models. Indeed, intratracheal delivery of Regnase-1-specific MOs attenuated acute lung injury and chronic pulmonary fibrosis by reducing pro-inflammatory cytokines in the lung, while the intracranial delivery of these molecules ameliorated symptoms of EAE, by promoting homeostatic microglial and T-cell responses. The clinical relevance is underscored by findings that Regnase-1 expression inversely correlates with disease severity in Multiple Sclerosis patients (117, 146).

Parallel approaches have been focused at restoring Regnase-1 by adeno-associated virus (AAV)-mediated gene therapy. This attempt is particularly relevant in conditions like PAH, where reduced Regnase-1 in alveolar macrophages is linked to disease pathogenesis. Myeloid-specific deletion of Regnase-1 in mice leads to spontaneous, severe PAH driven by unchecked IL-6 and Platelet-derived growth factor (PDGF) signaling, establishing a rationale for gene-based strategies to re-impose its protective, homeostatic function in the lung vasculature (116, 147). Conversely, strategic inhibition of Regnase-1 is being pursued to enhance adoptive cell therapies, such as chimeric antigen receptor (CAR)-T cell therapy. This approach is directly aligned with Regnase-1’s established role as an enforcer of T-cell quiescence. In CD8^+^ T cells and CAR-T cells, Regnase-1 degrades mRNAs critical for stem-like memory and persistence, including *T-cell factor 7* (*Tcf7* encoding TCF-1). Genetic deletion or knockdown of Regnase-1 “reprograms” these cells into long-lived, stem-like effector cells (termed TCF-1^+^ precursor exhausted T cells, or TPEX), which exhibit enhanced expansion, persistence, and cytotoxic efficacy in mouse models against solid tumors (melanoma) and leukemia. This effect is conserved in human CAR-T cells, where Regnase-1 deficiency promotes a TPEX phenotype and improves tumor clearance in a xenograft model of B-cell acute lymphoblastic leukemia, positioning Regnase-1 as a key target for engineering more potent and durable cell therapies (91, 148, 149).

Therefore, the translational toolkit for targeting the proteins belonging to the Regnase family is heterogenous and comprises oligonucleotides and gene therapy. However, efforts are underway to develop small-molecule or peptide that working as inhibitors can target the conserved NYN/PIN RNase domain, for instance by chelating its essential divalent metal ions or disrupting the adjacent CCCH zinc-finger motifs responsible for RNA binding, as elucidated by structural studies. Indirect approaches include modulating upstream signals that trigger Regnase-1 degradation or co-targeting other negative regulators like Protein Tyrosine Phosphatase, Non-Receptor Type 2 (PTPN2) to amplify the effects of Regnase-1 in the inhibition of T cells (26, 36).

In summary, the Regnase pathway represents a novel and promising frontier for therapeutic intervention in immune dysregulation and cancer. Its translational potential lies in context-specific strategies: enhancing the activity of some Regnase proteins via ASOs or gene therapy to suppress autoimmunity, fibrosis, and vascular inflammation, or selectively inhibiting Regnase proteins in adoptive immune cells to bolster antitumor immunity and T-cell persistence. However, these approaches carry inherent risks: systemic inhibition of Regnase-1—while boosting antitumor immunity—may precipitate autoimmune pathology, given that Regnase-1 deficiency in T cells and B cells is known to provoke fatal autoimmunity in mice (50). Similarly, chronic enhancement of Regnase-1 expression via ASOs or gene therapy could over suppress protective immune responses, increasing susceptibility to infections or impairing immunosurveillance. Moreover, the pleiotropic roles of Regnase proteins across tissues raise concerns about off-target effects; for instance, Regnase-1 overexpression in the heart exacerbates atrial fibrillation (111), highlighting the need for tissue-specific delivery systems. Finally, in the cancer context, inhibiting Regnase-1 could inadvertently fuel tumor-promoting inflammation or stromal activation, particularly in malignancies driven by NF-κB or IL-6. Beyond Regnase-1, emerging roles for Regnase-2 in glioblastoma suppression, Regnase-3 in interferon feedback and epithelial homeostasis, and Regnase-4 in lymphocyte activation offer new avenues for intervention. Future efforts must therefore expand from a Regnase-1-centric view to a family-wide perspective, developing paralog-specific modulators with spatiotemporal precision. Achieving this will require deeper mechanistic insight into each member’s target repertoire, regulatory networks, and tissue-specific functions. Ultimately, harnessing the Regnase pathway promises unprecedented control over immune responses, but success will depend on balancing potent immunomodulation with rigorous safeguards against autoimmunity, off-target inflammation, and loss of tissue homeostasis.
